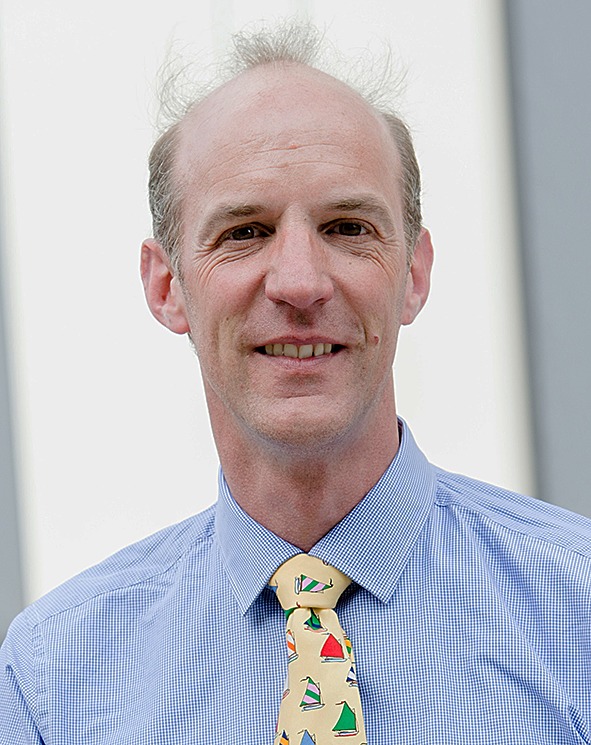# Letter from the editor

**DOI:** 10.1007/s00775-020-01792-1

**Published:** 2020-05-13

**Authors:** Nils Metzler-Nolte

**Affiliations:** Chair of Inorganic Chemistry I, Bioinorganic Chemistry, Faculty of Chemistry and Biochemistry, Ruhr University Bochum, Bochum, Germany

Dear JBIC Reader, dear colleague:

I am writing this Editorial in the middle of the Corona Crisis, like most of us working from my office desk at home. These are unprecedented times for most of us, who are lucky enough to never have experienced war, civil war, or similar hardships in our lifetimes. The virus has brought our scientific life to an almost complete shutdown, with labs closed all over the world, conferences canceled, travel suspended, exchange of students and researchers stalled, and research operations at large national and transnational facilities like synchrotrons being dramatically reduced. On the positive side, the need for serious, evidence-based science became painfully apparent to a public that would otherwise largely ignore, if not even joke about us. All of the sudden, scientists are regularly seen in evening news, and those governments that listen and follow their advice seem to fare better through the crisis. I am hoping that at least the respect for science will be long-lasting, as one of the possible positive outcomes of the crisis.

In these difficult times, JBIC continues operation as close to normal as possible. Not knowing how long regular research work in your labs will be suspended, I wish to highlight items from our editorial policy that may be particularly useful to remember:

1. JBIC welcomes submissions not only of original articles, but also of mini-reviews. A mini-review should be a critical summary of the current literature in a topical, not-too-specialized field of some general interest. It is typically between 5 and 15 printed pages in length, and has 50–150 references. A mini-review is not meant to be comprehensive, but rather a concise summary of the most important developments in the field. Highlighting current trends and including a critical perspective from the authors’ point of view is welcome. Mini-reviews can be submitted at any time without prior invitation, but you are most welcome to contact me informally to discuss scope and suitability of your intended submission. Of course, mini-reviews will undergo the same rigorous peer-review process as regular papers.
2. For JBIC, it is acceptable to involve coworkers such as experienced PhD candidates or postdocs in reviewing assignments, provided that the PI offers mentoring throughout the process, and ultimately assumes full responsibility for the submitted review. Of course, no conflict of interest must exist and anonymity must be observed as usual. It is advised to indicate the name of the coworker who helped with the review in the “Confidential Comments to the Editor” field during review submission.
3. We are very aware that many researchers face difficulties in meeting the timelines associated with revisions and the peer-review process during these times. Please contact me or your Associate Editor if you need additional time. The Editorial System will continue to send reminders automatically, but we promise to be flexible and try to accommodate your needs as good as possible.

Finally, and also on the bright side, this Issue features a “first” for me: In an *invited commentary*, Robinson and Glasfeld pick up on the topic of an article that appears later in the Issue (by Högbom et al.). Their commentary provides a background discussion on how metalloenzymes “pick” their metal, in a case where this is against the Irving–Williams series, and natural abundance. Generally, a commentary may refer to an article published in the journal. Or it may highlight important new methods or developments, or raise a discussion that is otherwise important for the Bioinorganic Community. Commentaries are short and opinions are welcome. I see them as an excellent way to engage the community in topical discussions in our field. Therefore, if you have a viewpoint or topic of general interest that you wish to share in the form of a commentary, please contact me directly and we will discuss the possibility.

And as all newsworthy items, your commentary will be highlighted on our Twitter account (@JBIC_Journal)—so follow the tweets. Enjoy reading!

Nils Metzler-Nolte.

April 2020.